# Markedly impaired bilateral coordination of gait in post-stroke patients: Is this deficit distinct from asymmetry? A cohort study

**DOI:** 10.1186/1743-0003-8-23

**Published:** 2011-05-05

**Authors:** Ronald Meijer, Meir Plotnik, Esther Groot Zwaaftink, Rob C van Lummel, Erik Ainsworth, Juan D Martina, Jeffrey M Hausdorff

**Affiliations:** 1 Rehabilitation Medical Centre Groot Klimmendaal, Department of Innovation, Research & Education, Room K009, PO Box 9044, 6800 GG Arnhem, Netherlands; 2 Research Department St. Maartenskliniek, Nijmegen, Netherlands; 3 Rehabilitation Medicine Department, University Medical Centre, Nijmegen, Netherlands; 4 Movement Disorders Unit, Department of Neurology, Tel Aviv Sourasky Medical Center, Tel Aviv, Israel; 5 Bar Ilan University, Ramat Gan, Israel; 6 McRoberts, The Hague, Netherlands; 7 Department of Physical Therapy, Sackler Faculty of Medicine, Tel Aviv University, Tel Aviv, Israel

## Abstract

**Background:**

Multiple aspects of gait are typically impaired post-stroke. Asymmetric gait is common as a consequence of unilateral brain lesions. The relationship between the resulting asymmetric gait and impairments in the ability to properly coordinate the reciprocal stepping activation of the legs is not clear. The objective of this exploratory study is to quantify the effects of hemiparesis on two putatively independent aspects of the bilateral coordination of gait to gain insight into mechanisms and their relationship and to assess their potential as clinical markers.

**Methods:**

Twelve ambulatory stroke patients and age-matched healthy adults wore a tri-axial piezo-resistive accelerometer and walked back and forth along a straight path in a hall at a comfortable walking speed during 2 minutes. Gait speed, gait asymmetry (GA), and aspects of the bilateral coordination of gait (BCG) were determined. Bilateral coordination measures included the left-right stepping phase for each stride φ_i_, consistency in the phase generation φ_CV, accuracy in the phase generation φ_ABS, and Phase Coordination Index (PCI), a combination of accuracy and consistency of the phase generation.

**Results:**

Group differences (p < 0.001) were observed for gait speed (1.1 ± 0.1 versus 1.7 ± 0.1 m/sec for patients and controls, respectively), GA (26.3 ± 5.6 versus 5.5 ± 1.2, correspondingly) and PCI (19.5 ± 2.3 versus 6.2 ± 1.0, correspondingly). A significant correlation between GA and PCI was seen in the stroke patients (r = 0.94; p < 0.001), but not in the controls.

**Conclusions:**

In ambulatory post-stroke patients, two gait coordination properties, GA and PCI, are markedly impaired. Although these features are not related to each other in healthy controls, they are strongly related in stroke patients, which is a novel finding. A measurement approach based on body-fixed sensors apparently may provide sensitive markers that can be used for clinical assessment and for enhancing rehabilitation targeting in post-stroke patients.

## Background

Among patients who experience a stroke, an altered gait pattern and impaired functional mobility are common, even at the conclusion of the typical rehabilitation process. Changes in gait post-stroke include reduced speed and increased energy expenditure. Gait asymmetry (GA) is also quite prevalent and is recognized as a key to understanding of the post-stroke deficits in gait and to improving the rehabilitation process in order to maximize mobility after a stroke [1,2]. However, a complete understanding of all of the factors that contribute to GA in post-stroke patients is lacking [2].

GA is only one aspect of bilateral activation of gait. When evaluating symmetry of walking, we address the question as to what extent the limbs perform similar walking movements. For example, one can compare the swing times performed by each leg. Usually, these measures are compared over series of steps and not per individual gait cycles [3]. Another feature is the timing of the left-right coordination of gait, namely the bilateral coordination of gait (BCG). This feature is distinctive from GA since it evaluates the level of coordination between the ongoing stepping movements of both legs. In other words: the individual performance of each leg is not evaluated but rather the interaction between their activation. Evaluating the left-right stepping phasing pattern (ideally 180°) is a convenient way to assess this interaction and is also done based on a series of steps.

These two aspects of bilateral activation of gait are not necessarily strongly correlated with one another nor are they simply synonymous terms [4,5]. In amputees, for example, the relative timing pattern of the gait cycle, the BCG, can remain constant while one leg will have much shorter swing times than the other, implying high asymmetry [6]. Consistent with the idea that these two properties are independent, only a weak correlation between GA and BCG was observed in patients with Parkinson's disease (PD)[4]; in these patients, unlike in amputees, a central nervous system asymmetric degenerative process likely leads both to increased GA and impaired BCG.

The present exploratory study was designed to investigate the nature of the relationship between BCG and GA in patients with hemiparesis due to stroke and to examine the potential clinical utility of measures based on BCG. For this purpose, we utilized recently introduced metrics of BCG that were found to be sensitive in other cohorts of subjects (e.g., elderly and young), but have not yet been applied to post-stroke patients [4]. In addition, we based our methods on body-fixed sensors (an accelerometer), an approach that could, theoretically, allow for easy implementation in clinical settings. We hypothesized that BCG and GA would both be impaired, compared to age-matched control subjects. Moreover, in contrast to what was observed in other populations, we speculated that impairment of BCG and increased GA are the result of the same underlying pathology in post-stroke patients, and, therefore, that these measures would be closely related to each other.

## Methods

### Study Participants

12 patients with hemiparesis due to stroke who underwent rehabilitation in the Groot Klimmendaal Medical Rehabilitation Centre (GKMRC), Arnhem, The Netherlands participated in this study. 12 age-matched healthy controls were recruited from a local fitness center. Inclusion criteria for the patients were: i) a stroke with a Motricity Index score of the paretic leg <100; ii) time since stroke: ≥ 1 month; iii) ability to safely walk 120 meters independently; iv) ability to follow simple instructions given in Dutch; v) and age range: 40-70 years. Exclusion criteria for the patients included: i) co-morbidity which might affect the walking pattern; ii) abnormal foot roll with absence of heel-strike at first ground contact (this may reflect a walking pattern with different characteristics, which would justify a separate research question); iii) and major psychiatric disorders or cognitive deficits. Inclusion criteria for the healthy adults were an observed normal walking pattern, no walking aids, absence of abnormalities of locomotor and neurological systems, and age between 40-70 years old. This study was approved by the human studies committee of the GKMRC. All participants provided informed written consent.

### Clinical Measures

To characterize the patient population, the Brunnstrom Fugl-Meyer Assessment Scale assessed functional motor recovery [7]. The Modified Ashworth scale measured muscle tone [8,9]. The Motricity Index evaluated strength [10,11]. The Berg Balance Scale provided a performance-based measure of postural control and balance [12]. The modified Nottingham Sensory Assessment evaluated the sensory function of the paretic foot[13] and the Achilles tendon reflex was used to examine possible plantar reflex (clonus). Use of assistive devices was also documented.

### Walking protocol

Before the execution of the walking test, each patient performed a practice walk to become acquainted to the test and the environment. The patients walked back and forth along a straight path at a self-selected, usual-walking speed along a quiet, level and well-lit 20 m long portion of a hall for 2 minutes (typically 4-6 times, for about 120 meters). Testing was performed without any aids, except for an AFO.

### Gait measurement

To measure the timing of the gait cycle over numerous strides, we used a tri-axial accelerometer (DynaPort MiniMod, McRoberts Inc.). The sensor was placed in a belt around the waist, attached at the level of the sacrum on the lower back, and measured gait cycle parameters via the McRoberts server [14-21]. The setup time for the measurement was approximately 2 minutes, including the preparation time for the patient. The post processing time was less than 5 minutes including uploading, calculation and reporting. In off-line analysis, only straight walking segments were included (the 180° turns at the corridor edges were excluded). The following parameters were extracted for each segment and averaged per subject across all segments (4-6 values per parameter per subject):

***Gait speed***segment length divided by the time to walk over those 20 meters

***Gait asymmetry (GA)***calculated as follows:(1)

where LSWT and RSWT represent each subject's mean value of the left and right swing times, respectively [4,22-25].

#### Phase Coordination index (PCI)

BCG is quantified by the PCI. This metric for quantifying the accuracy and consistency in generating left-right stepping phase is described in detail elsewhere [4,26]. Briefly, the stride and step-cycle times were determined from the accelerometer signal [20]. In addition, for each subject, we determined the leg with the long swing time and the leg with the short swing time based on the mean values. For each gait cycle, we first determined the left-right stepping phase for each stride φ_i _(ideally φ_i _= 180°):(2)

*t*_*Si*_and *t*_*Li*_are the times of heel-strike of step i of the short and long swing times, respectively, as *t*_*L(i+1)*_>*t*_*Si*_>*t*_*Li*__._[26]

To assess the consistency in the phase generation, we calculated the coefficient of variation of the mean of φ for each subject (φ _CV):(3)

in which δ is the standard deviation of *φ*, and  is the mean of the *φ*_*i*_s.

To assess the accuracy in the phase generation, i.e. how far is *φ *from the ideal of 180°, we calculated *φ *_ABS, the mean value of the series of absolute differences between the phase at each stride and 180°:(4)

The Phase Coordination Index (PCI) combines both quantities, the accuracy and consistency of the phase generation, according to the formula:(5)

where(6)

Thus PCI is described as a percent. A PCI value of 0 indicates "perfect" bilateral coordination, while values further away from 0 reflect increasingly impaired bilateral coordination.

### Statistical analysis

The Mann-Whitney U Test was used to compare demographic and gait parameters of the two groups. Spearman's rank correlation coefficients were determined to assess the associations between gait speed, GA and PCI. Summary measures are reported as mean ± standard error (SE). Statistical analyses were performed using SPSS 17.0. A p-value less than 0.05 was considered statistically significant.

## Results

Table 1 summarizes the demographic and clinical characteristics of the study participants. The relatively good scores on the Brunnstrom Fugl-Meyer Test (4.9 out of 6.0), the Motricity Index (82.6 out of 100.0), the Modified Ashworth Scale (0.9 out of 4.0), the Berg Balance Scale (53.4 out of 56.0) and the relatively high gait speed (1.1 m/sec) in the patients are likely a consequence of the inclusion criterion requirement of an ability to walk 120 meters. Regardless, they indicate that the patient population had only mild to moderate impairments in mobility. The mean number of steps/minute covered by patients and controls during the 2-minutes walking test was 100 (± 9), and 116 (± 11) respectively (p = 0.134). At home, six patients walked independently without any walking aids and six typically used a walking aid (cane, AFO, walker). During the walking test, except for the use of an AFO by two patients, the use of other walking aids was not allowed.

**Table 1:** Demographic and clinical parameters of the study groups (Means ± SEM)

Parameter	Stroke patients	Control subjects	P Value*
**Demographic**			

Age (years)	55.1 ± 1.8	56.2 ± 2.2	0.728

Gender (M/F)	6/6	5/7	0.999

**Clinical**			

Time since stroke (months)	6.9 ± 2.4	NA	

Side of paresis (left/right)	4/8	NA	

Brunnstrom Fugl-Meyer Assessment Scale **	4.9 ± 1.1 out 6	NA	

Modified Ashworth scale**	0.9 ± 1.0 out 4	NA	

Motricity Index for paresis **	82.6 ± 6.9 out 100	NA	

Berg Balance Test	53.4 ± 1.7 out 56	NA	

Sensory assessment paretic ankle: (intact/disturbed)	5/7	NA	

Achilles tendon reflex: (intact/disturbed)	7/5	NA	

* Mann-Whitney U Test; ** severity of stroke symptoms as observed in the hemiplegic leg; SEM- Standard Error of the mean; M- Male; F- female.

### Impairments in gait asymmetry and bilateral coordination of gait in stroke patients

**Figure 1:**
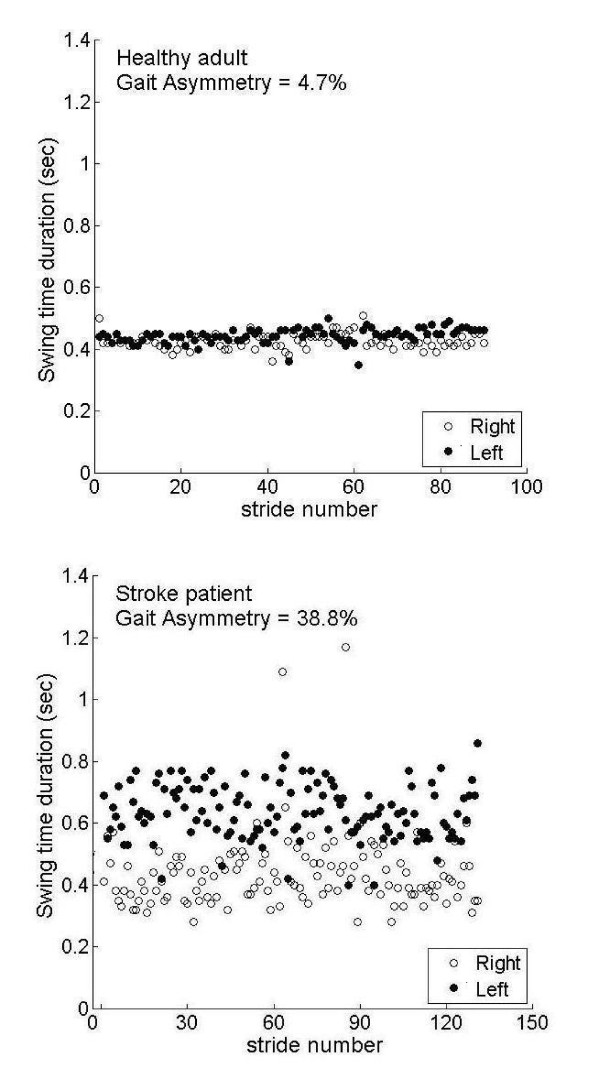
**a + b**: Left and right swing time values for all the strides of the two minute walk are shown for a healthy adult (figure 1a) and a patient (figure 1 b). Mean values of the right leg swing times were 0.47 seconds and 0.43 seconds for the control and stroke patient, respectively. The corresponding values for the left leg (paretic leg of the stroke patient) were 0.45 seconds and 0.64 seconds, respectively Healthy adult: mean number of steps/minute: 103; mean gait speed: 1.28 m/s. Stroke patient: mean number of steps/minute: 78.5; mean gait speed: 0.65 m/s. Both healthy adult and stroke patient had a number of steps/minute and gait speed in the bottom range of their groups (Table 2).

The gait of the stroke patients is characterized by an elongation in swing times in the paretic leg and increased GA (see Figure 1). Swing times of the left and right legs are plotted for the complete walking trial for a patient and control subject. For the control subject, swing values for the left and right leg virtually overlap. In contrast, for the patient with left hemiparesis, comparable swing values are seen only for the intact (right) leg and clear elongation in swing times is seen for the paretic (left) leg. Accordingly, GA is almost ten times higher for this stroke patient as compared to the control subject (see formula 1). The average value of GA in the patients was about 4 times larger than in the controls (see Table 2).

**Table 2:** Gait parameters of the study groups (Means ± SEM)

Parameter	Stroke patients	Control subjects	P Value*
Gait speed (m/sec)	1.1 ± 0.1	1.7 ± 0.1	<0.001

Steps/minute (number)	100 ± 9	116 ± 11	0.134

Short swing time percent (%)†	37.0 ± 1.0	38.2 ± 1.1	0.326

Long swing time percent (%)†	48.2 ± 2.6	40.4 ± 1.2	0.018

GA (%)	26.3 ± 5.6	5.5 ± 1.2	<0.001

φ (deg)	175.9 ± 6.9	182.2 ± 1.4	0.453

φ _ABS (deg)	23.1 ± 3.6	5.4 ± 1.1	<0.001

φ _CV (%)	6.7 ± 0.8	3.2 ± 0.4	0.002

PCI (%)	19.5 ± 2.3	6.2 ± 1.0	<0.001

* Mann-Whitney U Test; † Percent out of the whole gait cycle defined by this leg. SEM- Standard Error of the mean; PCI- Phase Coordination Index.

**Figure 2:**
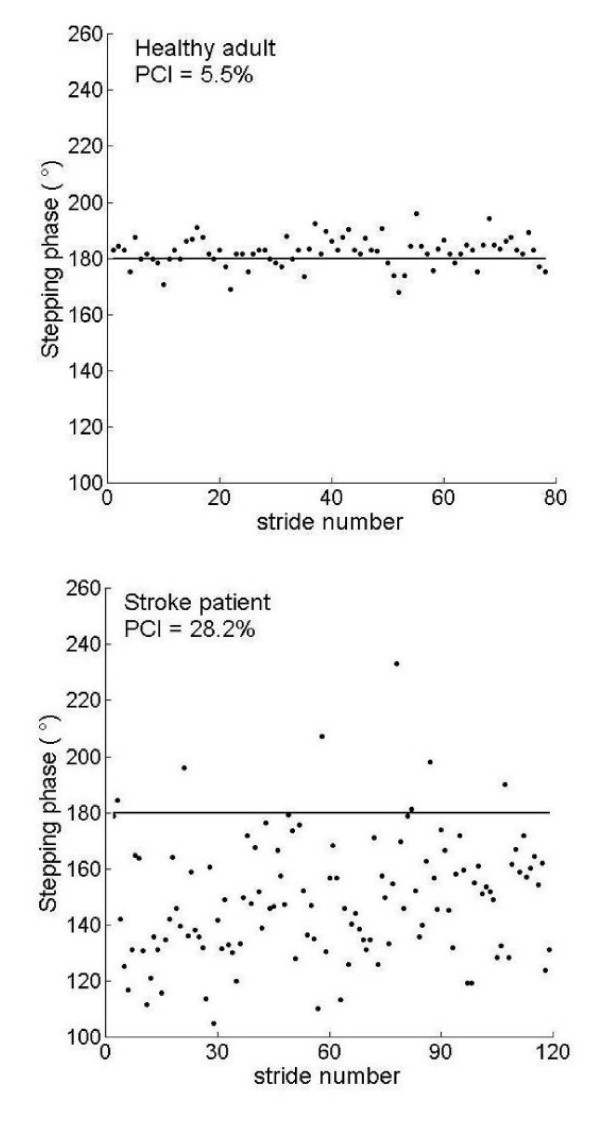
**a + b**: φ values for all the strides of the two minute walk are shown for a healthy adult (figure 2 a) and a patient (figure 2 b). PCI values are not dependent on the direction of deviation from the ideal 180° value (represented by solid line), i.e. higher or lower than 180°. Thus, group mean values of φ are close to 180° and are not statistically significantly different between the groups (Table 2), while φ_ABS, φ_CV, and thus PCI are highly increased in the patients. Healthy adult: mean number of steps/minute: 103; mean gait speed: 1.28 m/s. Stroke patient: mean number of steps/minute: 78.5; mean gait speed: 0.65 m/s.

In stroke patients, the left- right phasing coordination, the BCG, is characterized by both increased inaccuracy in generating anti-phased stepping and increased stride-to-stride inconsistency, as compared to the control group. This results in increased PCI values (Table 2 lower rows). Figure 2 illustrates this point. Stepping phase values are plotted for a representative healthy adult and a hemiplegic patient. Less scatter (high consistency) of φ and relative closeness (slightly above) to the ideal 180° line (high accuracy) characterize the gait of the control subject. In contrast, for the subject with hemi-paresis, phase values are loosely scattered and more distanced (below) from the ideal 180° line. All this results in about a 5 fold higher PCI value for this stroke patient. This example is consistent with the group findings; the average PCI was about 3 times larger in the patients, compared to the controls (recall Table 2).

Table 3 summarizes the associations among key gait parameters for the two groups. In both groups, gait asymmetry and PCI measures were not significantly associated with gait speed, consistent with the idea that these properties are independent of this general measure of walking abilities. In the healthy controls, PCI and GA were not related to each other. In contrast, in the stroke patients, a very strong association between the PCI and GA was observed.

**Table 3:** Spearman's ρ correlation values (p values in parentheses) for the relationships between gait speed, GA and PCI.

	Stroke Patients			Healthy Controls			
	Gait Speed	GA	PCI	Gait Speed	GA	PCI	

Gait Speed		-0.280 (0.379)	-0.266 (0.404)		-0.035 (0.914)	-0.322 (0.308)	Gait Speed

GA			**0.944 (<0.001)**			0.469 (0.124)	GA

## Discussion

The key findings of our investigation of BCG in post-stroke patients are that: A) kinematic variability related to BCG measures (*φ*_ABS, *φ*_CV, and PCI) is markedly higher in the stroke patients, compared to healthy controls, but not due to their slowed gait. As anticipated, gait speed was lower in the patients. However, whereas the patients' group mean gait speed was reduced by less than 50%, compared to the controls, patients' PCI values were generally 3 times larger. These relative differences support the idea that these BCG features of gait may be more sensitive to stroke than gait speed. B) BCG was strongly related to GA in the stroke patients, but not in the controls. To our knowledge, this is the first report to demonstrate that not only is gait asymmetric in stroke patients, but that a distinct property, the coordination of the left-right stepping phasing, is also clearly impaired in this patient population.

### Possible sources of the impaired left-right stepping coordination in post stroke patients

What is the source of the dis-coordination of left-right stepping seen in the present study? Impairments in bilateral coordination of rhythmic arm swinging in stroke patients were previously reported and attributed to instability of bilateral temporal coordination for this rhythmical task [27]. Imbalance in motor pathway integrity might lead to this instability [28]. The gait of healthy young adults who intentionally slow down is characterized by increased intra- and inter-limb variability [29]. The present study showed very low and statistically not significant correlations between gait speed and GA or PCI in both patients and controls, groups that walked at very different speeds. This suggests that these features of left-right symmetry and coordination are independent of walking speed (recall Table 3).

This possibility is consistent with the finding that leg-arm coupling was not related to gait speed in post-stroke patients [5]. Thus, while stroke patients walk slowly, this slowed gait pattern apparently is not the source of the mismatch between left-right stepping. At the same time, PCI was strongly correlated with GA, but only in the stroke patients. The lack of an association between PCI and GA in the control subjects supports the idea that an asymmetric gait is not necessarily an uncoordinated gait [4]. Regulation of temporal GA may be distinct from the rhythmic process of coordinating stepping in one leg with the other (ideally in an accurate 180° anti-phase pattern). Still, the question remains: why were GA and PCI so tightly coupled in the stroke patients?

Despite bilateral damage in stroke patients, in most cases, anatomical lesions are more extensive on one side of the brain [28]. Earlier studies on the relationship between sensorimotor impairments and gait asymmetry in patients with mild to moderate stroke found that symmetry of the swing phase duration between the two lower extremities was significantly related to a patient's status of motor recovery, regardless of the sensory status, and later it was suggested that spasticity of the ankle plantar flexors appeared to be the critical factor determining the temporal and spatial asymmetry of hemiplegic gait [30,31]. We speculate that hemiparetic stroke patients' asymmetric motor capabilities develop deficits in bilateral coordination because the motor commands are no longer equal for each leg. In addition, major sensory deficits impact the affected side in stroke patients, including diminished proprioception, one of the keys vital to locomotion coordination [32]. Thus, in stroke patients, the level of disease asymmetry may directly affect the level of coordination, and hence GA and PCI values will be correlated.

Compensatory mechanisms likely play a key role in the observed walking pattern [33]. Patients with Parkinson's disease (PD) usually suffer from asymmetric expression of disease-related motor symptoms, despite the fact that both cerebral hemispheres undergo neurodegeneration [23,25,34]. In contrast to the present findings, previous work demonstrated that PCI was only weakly correlated with GA in patients with PD [4]. Additional studies are needed to better understand why GA and PCI are so closely related in stroke patients.

### Clinical implications

The present findings underscore the notion that BCG is dramatically impaired in patients post-stroke and that BCG apparently plays an important role in the locomotion capacity of post-stroke patients, even among patients with only mild-to-moderate alterations in mobility (recall Table 1). This finding supports the recent recommendation to focus on gait symmetry in the rehabilitation of stroke patients[1] and would suggest that future rehabilitation interventions should take into account and specifically target left-right stepping coordination [35]. As noted above, simply focusing on gait speed, certainly an important indicator of functional ambulation abilities, will likely not be sufficient to optimally address bilateral coordination.

The present study also illustrates some of the advantages of using tri-axial accelerometry and the PCI metric. Subjects walked in conditions that are routinely found in a clinical environment. The accelerometer provided meaningful quantitative information regarding subtle gait features as well as robust discrimination between stroke patients and controls, without the need for relatively cumbersome gait analysis systems that restrict the measurements to specialized laboratory. Body-fixed accelerometry has the potential of expanding the assessment beyond the lab, to the at home and clinical settings [36,37]. Often, patients post-stroke prefer to regain a symmetrical walking pattern because of reasons related to appearance and self-image. Quantification of GA is very difficult to obtain using only visual observation or readily available clinical instruments. The objective metrics and sensitive markers described here could help to provide the patient and the therapist feedback about the alteration and progression of gait during the rehabilitation process and in response to different training protocols.

Swing time values of the leg which had the shorter swing times on average ('short swing') did not differ significantly between the stroke patients and healthy adults, while long swing time did (recall Table 2 and Figure 1). Clinicians often construe that gait asymmetry is caused by shortening the single support phase of the hemiplegic leg to compensate for the relative imbalance while standing on it. This would imply a shortening of the swing time in the non-affected leg. This may be so in case of a poor walking function after stroke with a slow gait speed [38]; the findings of the present study actually show that in stroke patients with relatively good walking function the single support phase is increased on the non-affected side (meaning longer swing times for the affected side), and that the single support time duration of the affected side remains the same as in healthy subjects. This may have implications for assessment and treatment.

### Study limitations and future directions

This exploratory study has several limitations. For example, the sample size was small. Larger scale studies are needed to confirm and build on these preliminary findings. Nonetheless, there was clearly sufficient power to observe highly significant group differences. Even in this group of patients with relatively mild disability (recall Tables 1 and 2), PCI values were markedly different from those observed in healthy controls and even from patients with Parkinson's disease [4]. In stroke patients who have more severe impairment and disability, PCI values may be exaggerated even further, suggesting that perhaps PCI-based metrics can be used to monitor therapy and recovery. Our study population was not representative for the whole post-stroke population, and the results cannot be generalized. Another limitation is the use of assistive devices. During the walking test session, patients were not allowed to use assistive devices except for an ankle foot orthosis. Half of the patients were accustomed to apply these devices during daily life. This implies a different walking pattern as walking without a device. A cane, for example, is known to affect asymmetry. To exclude carry over effects as much as possible, patients walked without the device during a practice test session before the start of the real walking test. Nonetheless, one could suggest that this study reflects the current bilateral abilities of patients post-stroke. Still, in future studies, it will be insightful to re-examine the associations between GA and BCG in patients with and without walking aids, to monitor potential changes in GA and BCG over time during the rehabilitation process until the moment the patients have apparently reached a plateau in their walking ability. Perhaps in these patients, the level of gait asymmetry will become correlated with gait speed [39]. In future studies, aspects of bilateral coordination should also be further investigated in other sub-types of stroke patients with a focus on the various primary symptoms to address questions such as: are impairments in BCG apparent and similar in patients with hemi-inattention? Another issue that warrants further research is the relationship between gait asymmetry and BCG and gait speed. We did not find such a relationship (recall table 3), but this question should be further addressed using within subject comparisons design in controls and in patients to probe the potential stabilizing effect of gait speed on these gait features. Mapping and monitoring BCG and GA and the relationship between these two features in diverse sub-groups of stroke patients may advance the understanding of mechanisms contributing to post-stroke gait deficits and in the selection and monitoring of rehabilitation strategies so that they can be tailored to the particular needs of a patient.

## Conclusions

In summary, this initial investigation of the relationship between GA and BCG in post-stroke patients demonstrates profound difficulties in the coordination of the anti-phase left-right stepping pattern that are apparently independent of gait speed. Additional work is needed to more fully explore the observed findings. Nonetheless, it appears that a small body-fixed, tri-axial accelerometer and a recently developed metric for assessing the bilateral coordination of gait (PCI) have the potential to enhance the quantitative monitoring of symptoms and the setting of rehabilitation goals in stroke patients.

## Competing interests

Two authors, RvL and EA, have a commercial interest, because they are employees of the firm that fabricates the accelerometry device. However, this did not have any influence on the content of the article.

## Authors' contributions

RM designed the study, supervised the collection of the data, supervised data-entry, performed data-analysis and interpretation, conducted the writing of the article and approved the final version of the article. MPl provided advice concerning the content, conducted the writing and approved the final version of the article. EGZ conducted the writing of the article and approved the final version of the article. RvL designed the study, supervised the collection of the data, supervised data-entry, performed data-analysis and interpretation, conducted the writing and approved the final version of the article. EA supervised data-entry, performed data-analysis, conducted the writing of the article and approved the final version of the article. JM provided the infrastructure, conducted the writing and approved the final version of the article. JH provided advice concerning the content, conducted the writing and approved the final version of the article.
